# Supplementary material for: Associations of metabolic syndrome and metabolically unhealthy obesity with cancer mortality: The Japan Multi-Institutional Collaborative Cohort (J-MICC) study
Source: PLoS One. 2022 Jul 8;17(7):e0269550. doi: 10.1371/journal.pone.0269550 (PMC9269937; doi:10.1371/journal.pone.0269550)
Supplement: Attachment — Submitted filename: ToEditorialOffice03162022.docx [file pone.0269550.s003.docx]

To Editorial Office, PLOS ONE

Thank you for your e-mail dated January 24, 2022 and very useful comments raised by the reviewers. We have modified the manuscript according to the comments. The changes and responses to the reviewer’s comments are as follows.

We hope that our revised version suitable for publication in the PLOS ONE.

Sincerely yours,

Kokichi Arisawa, MD, MSc, PhD

Department of Preventive Medicine,

Tokushima University Graduate School of Biomedical Sciences,

3-18-15, Kuramoto-cho, Tokushima 770-8503, Japan

（Responses to Editor and Reviewers）

Journal Requirements:

When submitting your revision, we need you to address these additional requirements.

1. Please ensure that your manuscript meets PLOS ONE's style requirements, including those for file naming. The PLOS ONE style templates can be found at

<https://journals.plos.org/plosone/s/file?id=wjVg/PLOSOne_formatting_sample_main_body.pdf> and

<https://journals.plos.org/plosone/s/file?id=ba62/PLOSOne_formatting_sample_title_authors_affiliations.pdf>

The manuscript was modified according to the PLOS One’s style requirements.

2. Please include your tables as part of your main manuscript and remove the individual files. Please note that supplementary tables (should remain/ be uploaded) as separate "supporting information" files.

Tables were included as part of the text. Supplementary tables 1 and 2 were attached as supporting information files.

3. Please include your full ethics statement in the ‘Methods’ section of your manuscript file. In your statement, please include the full name of the IRB or ethics committee who approved or waived your study, as well as whether or not you obtained informed written or verbal consent. If consent was waived for your study, please include this information in your statement as well.

In the Methods section, full ethics statement was added, including the names of the IRBs, the numbers, and written informed consent.

The research protocol was approved by the ethics committee of Nagoya University Graduate School of Medicine (IRB No. 2010-0939-7), Aichi Cancer Center Research Institute (IRB No. 2016- 2-10), Tokushima University Hospital (IRB No. 466-8), and all other institutions participating in J-MICC Study. During the survey, participants were informed that their participation was voluntary and written informed consent was obtained from all participants.

4. We note that the grant information you provided in the ‘Funding Information’ and ‘Financial Disclosure’ sections do not match.

When you resubmit, please ensure that you provide the correct grant numbers for the awards you received for your study in the ‘Funding Information’ section.

Name and number of grants were added to the Funding Information section.

5. In your Data Availability statement, you have not specified where the minimal data set underlying the results described in your manuscript can be found. PLOS defines a study's minimal data set as the underlying data used to reach the conclusions drawn in the manuscript and any additional data required to replicate the reported study findings in their entirety. All PLOS journals require that the minimal data set be made fully available. For more information about our data policy, please see <http://journals.plos.org/plosone/s/data-availability>.

Upon re-submitting your revised manuscript, please upload your study’s minimal underlying data set as either Supporting Information files or to a stable, public repository and include the relevant URLs, DOIs, or accession numbers within your revised cover letter. For a list of acceptable repositories, please see <http://journals.plos.org/plosone/s/data-availability#loc-recommended-repositories>. Any potentially identifying patient information must be fully anonymized.

Important: If there are ethical or legal restrictions to sharing your data publicly, please explain these restrictions in detail. Please see our guidelines for more information on what we consider unacceptable restrictions to publicly sharing data: <http://journals.plos.org/plosone/s/data-availability#loc-unacceptable-data-access-restrictions>. Note that it is not acceptable for the authors to be the sole named individuals responsible for ensuring data access.

We will update your Data Availability statement to reflect the information you provide in your cover letter.

The data used in the present study cannot be made publicly available, because the participants in the J-MICC Study have not given informed consent for public data sharing, and the ethics committee of the Tokushima University Hospital does not approve data sharing. At this time, interested researchers cannot receive access to the data. However, interested researchers can contact the Administration Office of the ethics committee of the Nagoya University Graduate School of Medicine ([iga-shinsa@adm.nagoya-u.ac.jp](mailto:iga-shinsa@adm.nagoya-u.ac.jp)). After receiving a request, informed consent from the participants will be sought.

6. One of the noted authors is a group or consortium “Japan Multi-institutional Collaborative Cohort (J-MICC) Study Group.” In addition to naming the author group, please list the individual authors and affiliations within this group in the acknowledgments section of your manuscript. Please also indicate clearly a lead author for this group along with a contact email address.

The name of the Japan Multi-Institutional Collaborative Cohort (J-MICC) Study was deleted from the authors. Instead, it was included in the title.

7. We note that you have included the phrase “data not shown” in your manuscript. Unfortunately, this does not meet our data sharing requirements. PLOS does not permit references to inaccessible data. We require that authors provide all relevant data within the paper, Supporting Information files, or in an acceptable, public repository. Please add a citation to support this phrase or upload the data that corresponds with these findings to a stable repository (such as Figshare or Dryad) and provide and URLs, DOIs, or accession numbers that may be used to access these data. Or, if the data are not a core part of the research being presented in your study, we ask that you remove the phrase that refers to these data.

Supplementary table 1 was added and the phrase “data not shown” was deleted.

8. Please include captions for your Supporting Information files at the end of your manuscript, and update any in-text citations to match accordingly. Please see our Supporting Information guidelines for more information: <http://journals.plos.org/plosone/s/supporting-information>.

Captions for supporting information files were added to the end of the text.

9. Please review your reference list to ensure that it is complete and correct. If you have cited papers that have been retracted, please include the rationale for doing so in the manuscript text, or remove these references and replace them with relevant current references. Any changes to the reference list should be mentioned in the rebuttal letter that accompanies your revised manuscript. If you need to cite a retracted article, indicate the article’s retracted status in the References list and also include a citation and full reference for the retraction notice.

Reference list was checked and modified.

[Note: HTML markup is below. Please do not edit.]

Reviewers' comments:

Reviewer's Responses to Questions

**Comments to the Author**

1. Is the manuscript technically sound, and do the data support the conclusions?

The manuscript must describe a technically sound piece of scientific research with data that supports the conclusions. Experiments must have been conducted rigorously, with appropriate controls, replication, and sample sizes. The conclusions must be drawn appropriately based on the data presented.

Reviewer #1: Yes

Reviewer #2: Yes

2. Has the statistical analysis been performed appropriately and rigorously?

Reviewer #1: No

Reviewer #2: I Don't Know

3. Have the authors made all data underlying the findings in their manuscript fully available?

The [PLOS Data policy](http://www.plosone.org/static/policies.action#sharing) requires authors to make all data underlying the findings described in their manuscript fully available without restriction, with rare exception (please refer to the Data Availability Statement in the manuscript PDF file). The data should be provided as part of the manuscript or its supporting information, or deposited to a public repository. For example, in addition to summary statistics, the data points behind means, medians and variance measures should be available. If there are restrictions on publicly sharing data—e.g. participant privacy or use of data from a third party—those must be specified.

Reviewer #1: Yes

Reviewer #2: Yes

4. Is the manuscript presented in an intelligible fashion and written in standard English?

PLOS ONE does not copyedit accepted manuscripts, so the language in submitted articles must be clear, correct, and unambiguous. Any typographical or grammatical errors should be corrected at revision, so please note any specific errors here.

Reviewer #1: No

Reviewer #2: Yes

5. Review Comments to the Author

Please use the space provided to explain your answers to the questions above. You may also include additional comments for the author, including concerns about dual publication, research ethics, or publication ethics. (Please upload your review as an attachment if it exceeds 20,000 characters)

Reviewer #1: The authors examined the associations of MetS and metabolically unhealthy obesity (MUHO) with cancer
mortality in a Japanese population.. Although the finding are potential, reviewer has few concerns:
- Table 1 is broken. Reviewer couldn't read the table.

Table 1 was included in the text.

- Consult the statistician to confirm the statistical tests used in the study.

Statistical methods used in this paper are commonly used ones, and our research team have sufficient experiences in epidemiologic research. In addition, data analysis was conducted by two independent persons, and it was confirmed that the figures in tables were identical.

- Check for the grammar and typo error.

The manuscript got English proofreading and errors were corrected.

- Please report the other medical underlying conditions and how they affect the finding? for instance asthma, diabetes.
- What were the other medications, patients were taking? Correlate them with the findings.

Other medications and histories of previous diseases were added to the text and the Table 1. These medications and previous diseases are as expected and may not have strong confounding effects on the results.

The proportions of those who had self-reported histories of colorectal polyps, fatty liver, high blood pressure, diabetes and dyslipidemia, and medication for high blood pressure, diabetes, and high blood cholesterol were higher, while histories of chronic gastritis and medication for constipation were less prevalent among participants with MetS than in those without MetS.

Reviewer #2: The author aimed to make a correlation with metabolic syndrome and cancer mortality, which in my view was successful. more and more studies of these sort need to be published reviewing the literature which gives a complete overview of the field for future scientists.

Thank you for your positive comments.

6. PLOS authors have the option to publish the peer review history of their article ([what does this mean?](https://journals.plos.org/plosone/s/editorial-and-peer-review-process#loc-peer-review-history)). If published, this will include your full peer review and any attached files.

If you choose “no”, your identity will remain anonymous but your review may still be made public.

**Do you want your identity to be public for this peer review?** For information about this choice, including consent withdrawal, please see our [Privacy Policy](https://www.plos.org/privacy-policy).

Reviewer #1: No

Reviewer #2: **Yes:** Ajay Palagani

[NOTE: If reviewer comments were submitted as an attachment file, they will be attached to this email and accessible via the submission site. Please log into your account, locate the manuscript record, and check for the action link "View Attachments". If this link does not appear, there are no attachment files.]

While revising your submission, please upload your figure files to the Preflight Analysis and Conversion Engine (PACE) digital diagnostic tool, <https://pacev2.apexcovantage.com/>. PACE helps ensure that figures meet PLOS requirements. To use PACE, you must first register as a user. Registration is free. Then, login and navigate to the UPLOAD tab, where you will find detailed instructions on how to use the tool. If you encounter any issues or have any questions when using PACE, please email PLOS at [figures@plos.org](mailto:figures@plos.org). Please note that Supporting Information files do not need this step.
